# How Having a Clear Why Can Help Us Cope With Almost Anything: Meaningful Well-Being and the COVID-19 Pandemic in México

**DOI:** 10.3389/fpsyg.2021.648069

**Published:** 2021-05-21

**Authors:** Angelica Quiroga-Garza, Ana C. Cepeda-Lopez, Sofía Villarreal Zambrano, Victor E. Villalobos-Daniel, David F. Carreno, Nikolett Eisenbeck

**Affiliations:** ^1^Department of Psychology, Universidad de Monterrey, Monterrey, Mexico; ^2^Department of Basic Sciences, School of Medicine, Universidad de Monterrey, Monterrey, Mexico; ^3^National Center for Disease Prevention and Control, Ciudad de México, Mexico; ^4^Department of Psychology, Universidad de Almería, Almería, Spain

**Keywords:** wellbeing, psychological distress, coping strategies, COVID-19 pandemic, perceived physical health, meaning-centered coping

## Abstract

**Background:** Coronavirus disease 2019 (COVID-19) has resulted in an increase in known risk factors for mental health problems. Mexico adopted lockdown and physical distancing as a containment strategy with potential consequences on day to day life, such as social isolation, loss of income and loneliness that can have important consequences in terms of mental health.

**Objective:** We aimed to examine the effect of the initial phases of the COVID-19 pandemic on psychological distress, well-being and perceived physical health among Mexican-base respondents and to examine whether coping strategies would play a potential intermediating role in relation to these variables. Under the Existential Positive Psychology perspective, an emphasis was made on meaning-centered coping.

**Methods:** A cross-sectional study was conducted between April 30 and June 16th 2020 among 604 Mexicans-base respondents of which 471 were women and 132 men. Data was collected by using online questionnaires. Psychological distress was measured using the Depression, Anxiety, and Stress Scale (DASS-21). The Brief COPE Inventory was used to assess problem-focused and emotion-focused coping strategies. We also used the Meaning-Centered Coping Scale (MCCS). PERMA-Profiler was used to assess well-being, perceived physical health, and loneliness. Profiler and Descriptive analyses and bivariate linear regression were performed to examine the association of variables.

**Results:** 45.9% of the participants reported moderate to extremely severe psychological distress. Our results demonstrate that problem-focused and emotion-focused coping were positively related to psychological distress, whereas meaning-centered coping was negatively associated with distress. Furthermore, psychological distress played a potential negative role in the perceived physical health, while meaning-centered coping and well-being buffered the negative influence of psychological distress on perceived physical health (completely standardized indirect effect = –0.01, SE: 0.012, 95% CI [−0.065; −0.017].

**Conclusion:** Meaning-centered coping was found to suppress the negative influence of psychological distress on sensation of decreased physical health corroborating the critical role of meaning in life in promoting well-being. Future studies can further examine the value of the critical role of meaning in life in promoting well-being as a protective factor against severe distress during traumatic events. Findings of this study can be used to orient policies and interventions aimed to alleviate suffering in the midst of the COVID-19 pandemic.

## Introduction

Those who have a “why” to live, can bear with almost any “how”. Viktor Frankl

In January 2020, the World Health Organization (WHO) declared the outbreak of a new coronavirus disease 2019 (COVID-19), to be a Public Health Emergency of International Concern. In March 2020, WHO made the assessment that COVID-19 can be characterized as a pandemic. On March 30, 2020, Mexico’s General Health Council declared a sanitary emergency due to COVID-19 pandemic (World Health Organization [WHO], 2020b). The following day, March 31, the Ministry of Health published an Administrative Ruling establishing the extraordinary actions to face the sanitary emergency generated by COVID-19, suspending all “non-essential activities,” and urging people in Mexico to stay at home until April 30 (Gobierno de México, 2020). In this context, the general population became vulnerable to the emotional impact of COVID-19 infection due to both the pandemic and its consequences worldwide.

The COVID-19 pandemic is having a profound effect on all aspects of society, including mental and physical health (Khan et al., 2020). Fear and anxiety about a new disease and what could happen can be overwhelming and cause strong emotions in adults and children. Public health actions, such as social physical distancing, can make people feel isolated and lonely and can increase stress and anxiety. However, COVID-19 pandemic, although instinctively may be considered undesirable, can also promote personal and spiritual growth (Wong, 2020). In recent years, new proposals by the existential positive psychology (PP 2.0; Wong, 2011) or second wave of positive psychology (Ivtzan et al., 2016; Lomas and Ivtzan, 2016), declare that in life, suffering is inevitable, but also potentially beneficial. This new paradigm of well-being claims that a meaning-centered coping style aimed to transform adversity into personal growth is crucial to transcend suffering (Wong, 2020).

Existential positive psychology (Wong, 2009) or PP 2.0 (Wong, 2011; Lomas and Ivtzan, 2016), was developed in an attempt to integrate humanistic existential psychology with positive psychology. PP 2.0 represents a development of the first wave of positive psychology (Seligman and Csikszentmihalyi, 2000), a paradigm criticized for being overly focused on positivity (e.g., Held, 2004; Wong and Roy, 2017). In addition to the positive qualities of human functioning proposed in the research of the first wave, PP 2.0 states that to get the best out of people it is necessary to accept the negative side of life. In life suffering is inevitable, but also potentially beneficial. According to this view, heartbreaking moments, trauma, death, illness, existential abyss, among others, although instinctively may be considered undesirable, can also promote personal and spiritual growth (Wong, 2011). As recently proposed by Wong (2020), the challenge of existential positive psychology is to catalyze a new science of well-being based on balance and wholeness by transcending the worse things in life and pursuing the best qualities in life. By learning how to achieve a dynamic balance between the positive and negative life forces in each context mature happiness and deep joy can be developed (Wong and Bowers, 2018).

In the face of adversity, there is evidence that discomfort and growth can coexist. Personal growth or “post-traumatic growth” is the result of greater personal strength, openness to new possibilities in life, greater connection with others, appreciation of life, and spiritual change (Calhoun and Tedeschi, 2006; Khanna and Greyson, 2015). Nevertheless, adversity does not guarantee growth. Post-traumatic growth depends on the coexistence of constructive functionality based on openness and acceptance; and illusory dysfunctionality based on self-deception or cognitive avoidance (Dempsey et al., 2000; Maercker and Zöllner, 2004; Zoellner and Maercker, 2006). Constructive coping and the severity of trauma-derived stress predict post-traumatic growth. As the acceptance of experience is greater potential to grow (Görg et al., 2017; Shipherd and Salters-Pedneault, 2018) and even the temporary illusory component can counteract emotional distress and promote self-consolidation (Zoellner and Maercker, 2006).

The way people relate to adversity is crucial for well-being. Coping abilities represent the individual set of mental and behavioral strategies adopted when facing traumatic experiences (Lazarus and Folkman, 1984). Coping abilities can determine the development of psychiatric diseases and maintaining of physical health, by reducing the risk of distress (Mikulincer and Solomon, 1989). Therefore, to cope well is to respond to the threat in ways that minimize its damaging impact, creating a sense of control (Bazrafshan and Delam, 2020). Most of the research literature indicates that people usually use two types of coping strategies to combat most stressful events: problem-focused and emotion-focused strategies. Problem-focused coping involves efforts to do something active to alleviate stressful circumstances (e.g., making action plans or seeking further information about the virus; Main et al., 2011; Cai et al., 2020; Gerhold, 2020). Emotion-focused coping strategies involve efforts to regulate the emotional consequences of stressful events (e.g., humor and reappraising difficulties to find benefits; Lazarus and Folkman, 1984), appraising the situations as controllable (Chee et al., 2020). The predominance of one type of strategy over another is determined, in part, by personal style and also by the type of stressful event. Maladaptive coping strategies, such as avoidance and minimization, have been associated with higher levels of distress (Theleritis et al., 2020) and significant number of psychological problems such as depression and anxiety (Blalock and Joiner, 2000), lower subjective well-being (Elliot et al., 2011), drug use (Carreno and Pérez-Escobar, 2019), and post-traumatic stress (Dempsey et al., 2000).

Acceptance of painful emotions and thoughts is one of the major areas in Existential Positive Psychology (Wong, 2011). However, this coping mechanism would be limited in terms of adaptability without an additional element: the use of meaning in life (Wong et al., 2006). Wong (1989) defines meaning in life as a socially and individually constructed system, which endows life with personal significance. The former reflects a status when individuals clearly identify the connection between themselves and the world, as well as a sense of realization, order, coherence, and purpose in the search and achievement of valuable goals in their lives (Reker and Wong, 1988; Steger et al., 2006). According to Folkman (2008) meaning-focused coping is in its essence, an appraisal-based coping in which the person draws on his or her beliefs (e.g., religious or spiritual), values (e.g., “mattering”), and existential goals (e.g., purpose in life) to motivate and sustain coping and well-being during a difficult time. However, the conceptualization of meaning-centered coping adopted in this work is based on Frankl’s (2014) approach to meaning and its ulterior development by Wong (2020) and Wong et al. (2006). Under this perspective, meaning-centered coping is understood as a set of attitudinal and behavioral strategies such as positive reframing, maintaining an attitude of hope and courage, life appreciation (also termed as “existential gratitude”, Jans-Beken and Wong, 2021), prosociality (self-transcendent values and actions), and engagement in other meaningful activities (see Wong, 2020).

Similarly, when it comes to physical and mental health (Batthyany and Russo-Netzer, 2014), meaning in life is one of the main components of well-being (Ryff, 1989) and is especially beneficial in adverse conditions (Wong, 2012; Hicks and Routledge, 2013). It has been associated with pain relief (Dezutter et al., 2015), disease risk reduction (Ryff, 2013; Ryff et al., 2016), prolongs hope in terminal diseases (Kállay and Miclea, 2007) and at the end of life (Wong, 2000; Heisel and Flett, 2014). Hence, meaningful interventions have shown improvements in quality of life and reduced psychological discomfort (Vos, 2016; Vos and Vitali, 2018). Particularly sources of relational sense, may play a key role in well-being by reducing depression (Carreno et al., 2020).

Based on the above-mentioned considerations, the current study sets out to examine the effect of the initial phases of the COVID-19 pandemic on psychological distress, perceived physical health and well-being among the Mexican population and to examine whether coping strategies would play a potential intermediating role in relation to these variables. This present research has three aims:

(1) To ascertain the level of psychological distress, perceived physical health and well-being among Mexican-base respondents during the initial phases of the COVID-19 pandemic.
(2) To examine the relationships between perceived physical health, psychological distress, well-being, and coping strategies (problem-focused, emotion-focused, and meaning-centered) among Mexican-base respondents; and
(3) To consider whether or not coping strategies mediates the relationship between psychological distress and perceived physical health.

## Materials and Methods

### Study Design

We conducted a cross-sectional study using data from the Mexican-base respondents that participated in a longitudinal multi-wave international online research on how people psychologically cope with the current COVID-19 crisis. Data for this study was collected during an intense period of the first lockdown in Mexico when restrictions were at their height. The study was approved by the Research, Biosafety and Bioethics Committees of the School of Medicine of the Universidad de Monterrey (Ref. CEI-EM UDEM 21/2020).

### Participants

Based on incidental, non-probabilistic sampling we recruited participants between April and June 2020 via social media. All participants were required to be 18+ years or older, currently resident in Mexico and able to read and write in Spanish. No other exclusion criteria were applied. Participation was voluntary and no incentives were provided.

### Measures

The survey was administered entirely online through the survey data collection platform Google Forms. The study was launched via a variety of social media platforms (Twitter, Facebook) and direct e-mail invitations during the first months (April, May, and June of 2020) of the COVID-19 health crisis. Potential participants were directed to study information via a weblink; those who wished to take part (after being reminded about their right to withdraw) were required to confirm that they met eligibility criteria and consented to take part.

### Individual Demographic Characteristics

We assessed gender, age, marital status, socio-economic status, education level, and population size of the town/city of the participants. Additionally, we asked the participants about the number of days they stayed at home during the lockdown and the number of people they are living in the same household with.

### Psychological Distress

We employed the Spanish version of Depression Anxiety and Stress Scale (DASS-21; Bados et al., 2005). Items are indicators of general psychological discomfort caused by distress–displacing emotional states–experienced over the past week and are classified on a 4-point Likert scale ranging from 0 (did not apply to me at all) to 3 (applied to me very much, or most of the time). In this study, both the subscales and the total scale showed satisfactory reliability (α total = 0.943; α stress = 0.88; α anxiety = 0.85; α depression = 0.88).

### Well-Being, Perceived Physical Health, and Loneliness

We used the Spanish version of the PERMA Profiler (Hernández-Vergel et al., 2018). The PERMA Profiler measures five domains of flourishing: positive emotion, engagement, relationships, meaning, and accomplishment. Each domain has three items, with the total score from these domains constituting a measure of well-being. The PERMA profiler also assesses perceived physical health (three items), negative emotion (three items, excluded from the study) and loneliness (one item; Cobo-Rendón et al., 2020). Each question offers a scale of seven points from 0 to 6, as indicated in each item to be consistent with the rest of the questionnaires in the package (see Dawes, 2008). Both measurements showed good internal consistency (well-being α = 0.829; physical health α = 0.831).

### Coping Strategies: Problem-Focused Coping, Emotion-Focused Coping and Meaning-Centered Coping

The Brief COPE Inventory is a 28-item self-report measure of coping styles in response to a stressful experience (Carver, 1997). We used the Spanish adaptation (Morán et al., 2010) of the Brief COPE Inventory to measure two different coping strategies: problem-focused and emotion-focused coping. Participants answer on a Likert-type scale of four response alternatives ranging from “0” (“I never do this”) to 3 (“I always do this”). Instructions were adapted to focus on coping in the context of COVID-19. Similar to previous studies on the psychological impact of pandemics (e.g., Yeung and Fung, 2007; Huang et al., 2020) we created two composite scores out of the 14 proposed subscales: problem-focused coping (active coping, planning, and instrumental support; α = 0.749) and emotion-focused coping (use of emotional support, self-distraction, relief, behavioral disconnection, positive reinterpretation, denial, acceptance, religion, substance use, humor, and self-blame; α = 0.791).

We used the meaning-centered coping scale (MCCS) recently developed by Eisenbeck et al. (2021) to measure meaning-centered coping during the current pandemic. Items describe coping strategies such as positive reframing, maintaining life appreciation and hope, adopting a courageous attitude against adversity, and being involved in prosocial and meaningful activities. Participants rated items on a Likert scale from 1 (I do not agree at all) to 7 (I completely agree). The instrument has shown satisfactory psychometric properties in 18 languages, including Spanish (detailed information can be shared upon request to the following email: nikolett.eisenbeck@ual.es). Consistency in this study was excellent (α = 0.899).

### Data Analytic Strategy

We first checked if the assumptions were met for all parametric tests conducted. We carried out the reliability analyses of the instruments and descriptive statistics of the collected data. In addition, we performed multiple correlations analyses (Pearson’s *r*) and regressions in order to subsequently analyze the interaction of the variables and to determine the explanatory mediation models. Based on the results, a sequential mediation analysis was performed to examine the interaction of variables using the computational tool macro PROCESS (model 4, bootstrapping 10,000 samples, 95% CI) statistical program for the social sciences (SPSS; Hayes, 2018). For this specific analysis, the perception of subjective discomfort was considered as the predictor variable (X), with subjective health perception as the output variable (Y), and subjective well-being (M) as the mediating variable. A second mediation analysis was carried out considering the same relationship between subjective discomfort as the predictor variable (X) and perceived physical health as the output variable (Y) with meaning-centered coping (M) as the mediator variable. Subsequently, a new analysis was carried out to conduct sequential mediation with meaning-centered coping as the first mediator (M1) and subjective well-being as a second mediator (M2) in the ratio of the predictor variable (X), subjective discomfort, and perceived physical health as the result variable (Y).

## Results

The study population consisted of 604 Mexican residents between the ages of 18–80 years, who participated on a voluntary basis (*M* = 41.89; *SD* = 13.72). Table 1 shows the sociodemographic characteristics of the study participants. The majority of respondents were female (78%), single (22.3%), from Northern Mexico (76.7%) with a household size of >3 people (81.7%). Of the sample, 51% reported a socioeconomic status above average, 47.2% below average and 1.8% average.

**TABLE 1:** Sociodemographic variables and frequencies in the participants (*n* = 604) of the study on the psychological effects of the COVID-19 pandemic on the Mexican population.

**Variable**		**Frequency (*n*)**	**Percentage (%)**
Gender	Female	471	78.0
	Male	132	21.9
Age (years)	18–20	24	4.0
	21–30	122	20.2
	31–40	156	25.8
	41–50	115	19.0
	More than 50	187	31.0
Economic status	Above average	308	51.0
	Average	11	1.8
	Below average	285	47.2
Geographic region	Northwest	10	1.7
	Northeast	453	75.0
	West	56	9.3
	Center	62	10.3
	Southeast	23	3.8

We found severe to extremely severe psychological distress in 16.4% of the study participants and moderate to severe psychological distress in 29.5%. Table 2 presents the univariate correlations between the main variables. To recap, a higher score on psychological distress indicated worse levels on perceived physical health [*r*_(__604__)_ = −0.270, *p* < 0.001]. The analyses revealed that higher levels of psychological distress were associated with problem-focused coping [*r*_(__604__)_ = 0.238, *p* < 0.001] and emotion-focused coping [*r*_(__604__)_ = 0.415, *p* < 0.001]. Well-being was positively correlated with perceived physical health [*r*_(__604__)_ = 0.480, *p* < 0.001] and meaning-centered coping [*r*_(__604__)_ = 0.616, *p* < 0.001].

**TABLE 2:** Correlation matrix for variables in the study (*n* = 604).

**Variables**	**1**	**2**	**3**	**4**	**5**	**6**
(1) Psychological distress	1	−0.270^**^	−0.363	0.238^**^	0.415^**^	−0.163^**^
(2) Perceived physical health		1	0.480^**^	0.090^*^	−0.004	0.300^**^
(3) Well-being			1	0.286^**^	0.085^*^	0.616^**^
(4) Problem-focused coping				1	0.573^**^	0.395^**^
(5) Emotion-focused coping					1	0.280^**^
(6) Meaning-centered coping						1

^*^Correlation is significant at the 0.05 level (2-tailed). ^**^Correlation is significant at the 0.01 level (2-tailed).

Multiple regression analyses were carried out to determine the predicted value of the variables psychological distress, well-being and coping strategies on perceived physical health. The results indicated that well-being [β = 0.433 (0.422; 0.662) *p* < 0.001] and psychological distress [β = 0.109 (−0.341; −0.038) *p* = 0.014] explained 23.5% of the perceived physical health (Table 3). A second model including only the three coping strategies, problem-focused coping, emotion-focused and meaning-centered coping could explain 9.4% of the perceived physical health. Meaning-centered coping [β = 0.320 (0.223; 0.380) *p* < 0.001] was a positive predictor of perceived physical health (see Table 4).

**TABLE 3:** Predictive regression model for subjective perception of health of participants (*n* = 604).

*F*_(_*_5_,_603_*_)_ = 38.1**, $R_{a}^{2}=0.235$				
**Predictor variables**	**β**	***t***	***p***	**95% *CI***
Subjective well-being	0.433	8.846	<0.001	[0.422; 0.662]
Psychological distress	−0.109	−2.455	0.014	[−0.341; −0.038]
Problem-focused coping	−0.023	−0.496	0.620	[−0.187; 0.111]
Emotion-focused coping	0.011	0.242	0.809	[−0.213; 0.273]
Meaning-centered coping	0.021	0.437	0.662	[−0.069; 0.109]

**TABLE 4:** Subjective health perception regression model with coping types as predictive variables (*n* = 604).

*F*_(_*_3_,_603_*_)_ = 21.9**, $R_{a}^{2}=0.094$				
**Predictor variables**	**β**	***t***	***p***	**95% *IC***
Problem-focused coping	0.026	0.518	0.604	[−0.117; 0.200]
Emotion-ffocused coping	−0.109	−2.291	0.022	[−0.528; −0.041]
Meaning-centered coping	0.320	7.568	<0.001	[0.223; 0.380]

**FIGURE 1:**
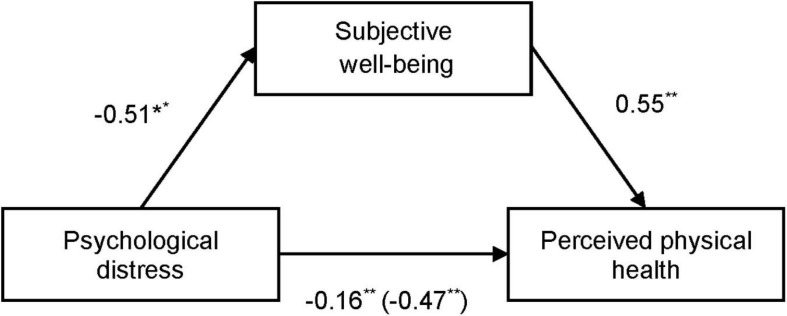
Mediational analysis of subjective well-being in the relation between psychological distress and perceived physical health. Direct effect after including the mediator is in brackets. ^**^p ≤ 0.001; ^*^p ≤ 0.05.

**FIGURE 2:**
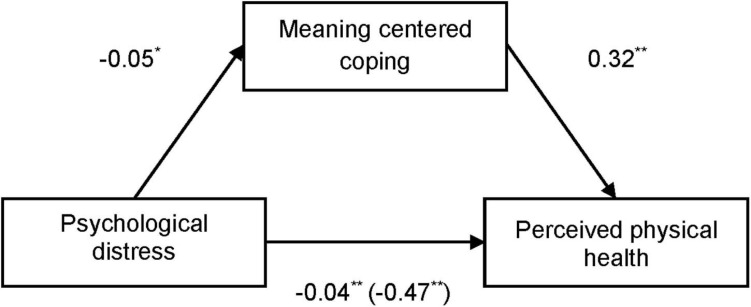
Mediational analysis of meaning cantered coping in the relation between psychological distress and perceived physical health. Direct effects after including the mediator are in brackets. ^**^p ≤ 0.001; ^*^p ≤ 0.05.

**FIGURE 3:**
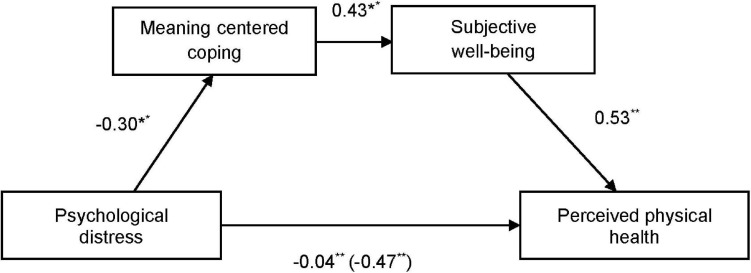
Sequential mediation analysis of meaning centered coping (serial mediator 1) and subjective well-being (serial mediator 2) in the relation between psychological distress and perceived physical health. Direct effects after including the mediator are in brackets. ^**^p ≤ 0.001; ^*^p ≤ 0.05.

We examined the potential mediating role of psychological distress and meaning-centered coping on perceived physical health. Figure 1 shows how the negative direct effect of psychological distress on perceived physical health [−0.47, SE 0.069, 95% CI (−0.606; −0.337)] was mediated by subjective well-being [completely standardized indirect effect −0.016, SE 0.021, 95% CI (−0.202; −0.119)]. Moreover, Figure 2 shows how meaning-centered coping also mediated the effect of psychological distress on perceived physical health [completely standardized indirect effect −0.043, SE 0.013, 95% CI (−0.072; −0.019)]. Finally, a sequential mediation analyses shows the interaction of meaning-centered coping and subjective well-being on the direct effect of psychological distress on perceived physical health [completely standardized indirect effect −0.04, SE 0.012, 95% CI (−0.065; −0.017)] (Figure 3).

## Discussion

This cross-sectional study shows that during the initial phases of the COVID-19 pandemic crisis, almost half of the Mexican-base respondents reported moderate to extremely severe psychological distress. Overall, psychological distress had a negative association with the perceived physical health of the study participants, while meaning-centered coping and well-being seem to buffer the negative influence of psychological distress on perceived physical health. Faced with the current landscape in Mexico where the impact of the COVID-19 pandemic keeps vulnerable people confined at home, the findings of this study are encouraging.

The current study is among the first to corroborate the critical role of coping strategies centered around meaning in life rather than around problem-solving within the context of the COVID-19 pandemic among Mexican-based respondents. It has been proposed that when individuals face uncontrollable situations such as this pandemic, meaning can contribute to individuals’ adjustment maintaining some level of well-being (Frankl, 1984; Wong, 2010, 2011). Thus, our findings are consistent with this theory on meaning-centered coping mediating the negative influence of psychological distress on perceived physical health. Our results are also in accordance with other studies, which indicate that during adverse life events meaning-centered coping can integrate cognitive, emotional, behavioral, personal and interpersonal elements that could influence subjective well-being with a sequential positive impact on the perception of physical health despite the presence of psychological distress (Wong, 2012; Van Tongeren et al., 2016; Eisenbeck et al., 2021; Jans-Beken and Wong, 2021).

In light of this, coping strategies aimed to promote and sustain meaning in life definitely require more attention. In addition, we are proposing *meaningful well-being* as the mechanism through which a meaning-centered approach to day to day vicissitudes exert a positive influence on well-being. In line with eudaimonic well-being based on meaning of person’s life at a given moment, that is, having the courage at all times particularly in adversity (Frankl, 1984, 2004, 2014), focusing on resources and strengths (Epston and White, 1992), investing significant effort (Waterman et al., 2010), and how feelings of mastery have an influence on health (Diener et al., 2017). Further research must follow.

The biopsychosocial mechanisms underlying the positive influence of meaning-centered coping on resilience to psychological distress are still understudied. The protective effect of meaning-centered coping on maintaining perceived physical health, may moderate genetic and environmental vulnerabilities and confer resilience to psychological distress. Recent research has found that chronic social adversity activates a conserved transcriptional response to adversity (CTRA), characterized by up-regulated expression of pro-inflammatory genes and decreased expression of antiviral- and antibody-related genes (Cole et al., 2015). Instead, eudaimonic well-being, which is centered on meaning in life, may act as a buffer against the adverse impact of social adversity on the CTRA gene expression (Cole et al., 2015; Fredrickson et al., 2015; Boyle et al., 2019). It has been proposed that meaning making can help to prevent a potential negative cycle in which intrusions both provoke and are triggered by chronic physiological arousal and emotional distress (McFarlane, 1992). There is a need for the discovery of mechanistically driven interventions to address the psychological, social, and neuroscientific aspects of this pandemic. Future research could explore if meaning-centered coping could moderate the potential negative physiological distress via its effects on the hypothalamic-pituitary-adrenocortical system, the noradrenergic system, and central oxytocin pathways.

Many of the coping mechanisms appear to be beneficial depending on the particular situation and context. A large body of research has examined the relationship between problem-focused coping and its psychological outcomes; however, the findings are mixed. In health workers (Cai et al., 2020), older adults (Gerhold, 2020) and in the general population (Guo et al., 2020) problem-focused coping has proven to lessen depression, while other studies on health professionals in China (Huang et al., 2020) and Italy (Vagni et al., 2020) have found increased anxiety, anger and fear that could be associated with limited understanding of the task that hinders effective decision-making. Moreover, problem-focused coping was found to be predominantly helpful in high controllability situations, while emotion-focused coping was more effective under low controllability conditions (Baker and Berenbaum, 2011). COVID-19 is an unpredictable situation, with high levels of uncertainty where problem-focused strategies might have limited utility. The end of the COVID-19 crisis feels remote, as promised treatments or vaccines will not be available for months at least, strategies that allow restructuring the emotional response, such as meaning-centered coping, can mediate the impact of distress, similar to what happens when dealing with cancer diagnosis (Jim and Andersen, 2007).

In our study, problem-focused coping and emotion-centered coping were associated with higher psychological distress. Findings in our study are similar to those in nurses (Huang et al., 2020) and health-care workers (Man et al., 2020) who tend to increase unpleasant emotional expression such as panic and sadness the more they face the pandemic. In our study, this could be related to increased fear due to the effect of repeated media consumption about COVID-19.

The findings from the DASS-21 identified that many of the participants (45.9%) reported moderate to extremely severe psychological distress. These finding adds to a growing body of evidence that shows how COVID-19 pandemic is having monumental effects on the mental health and well-being of populations worldwide (Holmes et al., 2020). The changes to daily life as a result of lockdown restrictions has added stress to many individuals’ lives (e.g., increased physical isolation and loneliness, closure of schools forcing parents to home-school their children while working from home themselves and widespread job losses). Such disruption to normal routine, activities and livelihoods is having monumental effects on the mental health and well-being of populations worldwide (World Health Organization [WHO], 2020a). Our results are similar to a study perform among Mexican-base respondents during the similar period of time. In that study 50.3% of the respondents rated the psychological distress of COVID-19 outbreak as moderate to severe (Cortés-Álvarez et al., 2020). However, it is noteworthy that the prevalence of psychological distress reported here is greater than that reported in China (Wang et al., 2020). The later could be explain by the fact that information on how the virus spreads and how it was affecting people around the world (infectability and lethality) preceded its arrival to Mexico. A recent survey performed by the WHO in 130 Member States revealed that 89% (116) of the countries had mental health and psychological support as part of their national COVID-19 response plans, however, only 17% said they had committed additional funding for this (World Health Organization [WHO], 2020c). It is unclear how we will deal with this looming mental health crisis, especially in low-income and middle-income countries like Mexico.

Besides the contribution mentioned above, the study has some limitations. The administration of the questionnaire was via electronic support (to which not everyone was familiar). Our study is also limited by incidental sampling and by not achieving balanced participation, e.g., by perceived socioeconomic status or by geographical region. A further limitation is the use of self-report measures which are prone to socially desirable answers. Also, the cross-sectional design of this study cannot establish a cause–effect relationship between lockdown measures the development of psychological distress, physical well-being and the different types of coping strategies. Any generalization to other populations should be made with caution. Finally, problem-focused and emotion-focused coping were measured as a two general sets composed of different coping strategies. Several of these strategies are clearly maladaptive (e.g., use of substances, denial, self-blame), which can explain the negative relation of these coping styles with well-being. Future studies should explore which specific problem-focused and emotion-focused strategies are maladaptive or adaptive in the present pandemic.

To sum up, the results of this study demonstrate that, during the initial phases of the COVID-19 pandemic crisis, almost half of the Mexican-base respondents reported moderate to extremely severe psychological distress. Meaning-centered coping was found to suppress the negative influence of psychological distress on sensation of decreased physical health corroborating the critical role of meaning in life in promoting well-being. Meaning-centered coping may help individuals learn how to cope in the face of an extremely traumatic and uncontrollable events. Future experimental interventions are needed to clearly demonstrate how various sources of meaning contribute to well-being. Finally, more research is needed to further understand the critical role of meaning in life in promoting well-being as a protective factor against severe distress especially during traumatic events.

## Data Availability Statement

The raw data supporting the conclusions of this article will be made available by the authors, without undue reservation.

## Ethics Statement

The studies involving human participants were reviewed and approved by Research, Biosafety, and Bioethics Committees of the School of Medicine, Department of Health Sciences of the Universidad de Monterrey (Ref. CEI-EM UDEM 21/2020). The patients/participants provided their written informed consent to participate in this study.

## Author Contributions

DC and NE generated the idea. AC-L, AQ-G, and SV conducted the study. AQ-G and AC-L wrote the first draft of the manuscript. All authors critically revised it for important intellectual content, gave final approval to the finished manuscript, and agreed to be accountable for all aspects of the work.

## Conflict of Interest

The authors declare that the research was conducted in the absence of any commercial or financial relationships that could be construed as a potential conflict of interest.
